# Antimicrobial Peptides in Reptiles

**DOI:** 10.3390/ph7060723

**Published:** 2014-06-10

**Authors:** Monique L. van Hoek

**Affiliations:** National Center for Biodefense and Infectious Diseases, and School of Systems Biology, George Mason University, MS1H8, 10910 University Blvd, Manassas, VA 20110, USA; E-Mail: mvanhoek@gmu.edu; Tel.: +1-703-993-4273; Fax: +1-703-993-7019

**Keywords:** antimicrobial peptides, antibacterial, reptile, biofilm, broad-spectrum, Gram-positive, Gram-negative

## Abstract

Reptiles are among the oldest known amniotes and are highly diverse in their morphology and ecological niches. These animals have an evolutionarily ancient innate-immune system that is of great interest to scientists trying to identify new and useful antimicrobial peptides. Significant work in the last decade in the fields of biochemistry, proteomics and genomics has begun to reveal the complexity of reptilian antimicrobial peptides. Here, the current knowledge about antimicrobial peptides in reptiles is reviewed, with specific examples in each of the four orders: Testudines (turtles and tortosises), Sphenodontia (tuataras), Squamata (snakes and lizards), and Crocodilia (crocodilans). Examples are presented of the major classes of antimicrobial peptides expressed by reptiles including defensins, cathelicidins, liver-expressed peptides (hepcidin and LEAP-2), lysozyme, crotamine, and others. Some of these peptides have been identified and tested for their antibacterial or antiviral activity; others are only predicted as possible genes from genomic sequencing. Bioinformatic analysis of the reptile genomes is presented, revealing many predicted candidate antimicrobial peptides genes across this diverse class. The study of how these ancient creatures use antimicrobial peptides within their innate immune systems may reveal new understandings of our mammalian innate immune system and may also provide new and powerful antimicrobial peptides as scaffolds for potential therapeutic development.

## 1. Introduction

Reptiles are among the oldest amniotes. They are cold-blooded (ectothermic) vertebrates with dry and scaly skin that usually lay soft-shelled eggs with amniotic membranes. They thrive in diverse environments, ranging as far north as Hudson’s Bay in Canada, and as far south as Cape Horn, Chile. They range from very small geckos to enormous crocodiles and have survived millennia of evolution. They are highly adapted to their environment, including their innate immune systems, allowing them to be such successful animals. Antimicrobial peptides are part of the innate immune system, and may contribute to the survival of these animals in microbe-filled, challenging environment. This paper will review the known and predicted antimicrobial peptides of reptiles. A set of known host defense antimicrobial peptides is collected in the Antimicrobial Peptide Database [1,2] and the examples from reptiles have been annotated (Table 1). Recently, many reptilian genomes have been sequenced. From these genomes, potential antimicrobial peptide genes have been predicted here by bioinformatics analysis (Table 2, Table 3, Table 4, Table 5, Table 6, Table 7 and Table 8), which should be synthesized and tested for activity in future work. Overall, the Reptile class thrives in diverse and challenging environments, partly due to their robust innate immune system, and our hypothesis is that antimicrobial peptides contribute to the evolutionary success of reptiles as they do in mammals and other species.

**Table 1:** Known reptile antimicrobial peptides identified in the Antimicrobial Peptide Database (APD2) [2].

Peptide name	Sequence	APD Identified	Source Organism	Comment Reference	Activity (*)
**Cathelicidin**					
OH-CATH	KRFKKFFKKLKNSVKKRAKKFFKKPRVIGVSIPF	AP00895	*O. Hannah* (Snake)	Derivatives: OH-CATH30; OH-CM6 [3,4]	G+, G−
Derivative OH-CATH30	KFFKKLKNSVKKRAKKFFKKPRVIGVSIPF			[5,6]	G+, G−
Derivative OH-CM6	KFFKKLKKAVKKGFKKFAKV			[3, 4]	G+, G−
BF-CATH	KRFKKFFKKLKKSVKKRAKKFFKKPRVIGVSIPF	AP00896	*Bungarus fasciatus* (Snake)	[7]	G+, G−, F, Cancer cells
Derivative BF-30	KFFRKLKKSVKKRAKEFFKKPRVIGVSIPF	AP01239	*B. fasciatus*	[8,9,10]	G+, G−
Derivative BF-15	KFFRKLKKSVVKRFK		*B. fasciatus*	[8]	G+, G−
NA-CATH	KRFKKFFKKLKNSVKKRAKKFFKKPKVIGVTFPF	AP00897	*N. atra* (Snake)	[3]	G+, G−
**Waprin**					
Omwaprin	KDRPKKPGLCPPRPQKPCVKECKNDDSCPGQQKCCNYGCKDECRDPIFVG	AP01589	*Oxyuranus* *microlepidotus* (Snake)	4S = S [11, 12]	G+
**Proline Rich**					
Lethal peptide I/Waglerin	GGKPDLRPCHPPCHYIPRPKPR	AP00238	*Trimeresurus wagleri*, Wagler’s pit viper (Snake)	P24335, [13]	Tx
**β-Defensin or defensin-like**					
TBD-1 (Turtle β-defensin 1)	YDLSKNCRLRGGICYIGKCPRRFFRSGSCSRGNVCCLRFG	AP01380	Turtle	3S = S [14]	G+, G−, F
Pelovaterin	DDTPSSRCGSGGWGPCLPIVDLLCIVHVTVGCSGGFGCCRIG	AP01381	Turtle	2JR3 BBBh2o [15, 16]	G−
TEWP (turtle egg-white protein)	EKKCPGRCTLKCGKHERPTLPYNCGKYICCVPVKVK	AP01382	Turtle	2B5B XXQ [17,18,19]	G-, V
**Crotamine defensin-like toxin**					
Crotamine	YKQCHKKGGHCFPKEKICLPPSSDFGKMDCRWRWKCCKKGSG	AP01650	Snake Venom	1Z99, 3S = S ZZP [20, 21]	G+, G−, F, P, Mammalian and Cancer cells

(*) Activity abbreviations: G+, G−, inhibiting both G+ and G− bacteria; G+, Gram-positive bacteria only, G−, Gram-negative bacteria only; V, antiviral; F, antifungal; P: antiparasitic, Tx: Toxin activity to mammals.

## 2. Four Orders of Reptiles

The structural and sequence diversity of antimicrobial peptides is impressive (see below), but it is useful to first review the phylogenetic and physiological diversity of the Reptilia class in order to appreciate all the ecosystems and environments that they live in and the prey that they eat.

**Figure 1:**
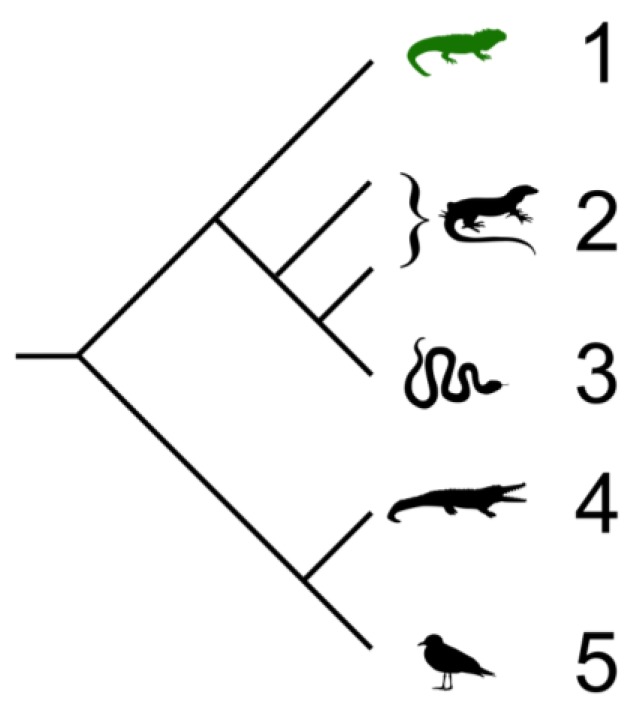
Cladogram showing the relationships of extant members of the Sauria (Sauropsida) which includes birds and reptiles. Branch lengths are not representative of divergence time. 1. Tuataras; 2. Lizards; 3. Snakes; 4. Crocodiles; 5. Birds. Cladogram by Benchill, licensed under the Creative Commons Attribution 3.0 Unported license [24].

All living reptiles fall within the kingdom Animalia, the phylum Chordata, the class Reptilia, and the clade Sauropsida [22]. There are four orders within the class Reptilia: turtles and tortoises (Testudines), tuataras (Sphenodontia), snakes and lizards (Squamata), and crocodilans (Crocodilia). Each order will be briefly introduced below. Many reptile species are endangered, including the painted turtle, and the Siamese crocodile. Although considered by phylogenetic analysis to be “monophyletic” with avians (Sauropsida; See Cladogram, Figure 1), reptiles are still generally considered to be separate from birds. Some researchers use the term avian reptile and non-avian reptile to distinguish the two groups [23]. Within the non-avian reptilians, the crocodilians are considered to be the most closely related to the avian branch. Interestingly, this evolutionary connection is reflected in the antimicrobial profile in that neither avians nor reptilians encode α-defensin antimicrobial peptides, for example, which are a critical part of mammalian innate immunity. In contrast, avians do not appear to express hepcidin peptides, while reptiles do, highlighting a potential difference.

### 2.1. Testudines (Turtles and Tortises)

The testudine order includes the turtles and tortosises. The western painted turtle (*Chrysemys picta bellii*) is a small turtle of North America, commonly found in ponds and other slow-moving fresh water [25].

**Figure 2:**
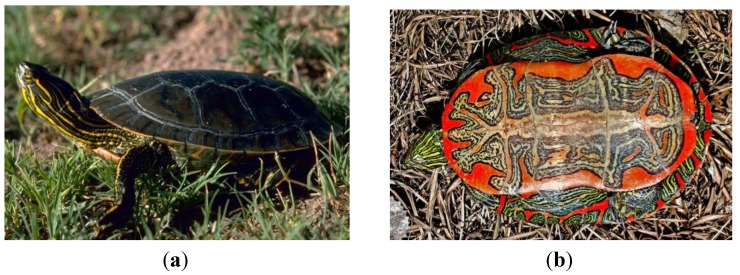
Western Painted Turtle *Chrysemys picta bellii*. (**a**) Western painted turtle. Photo by Gary M. Stolz, U.S. Fish and Wildlife Service in the Public domain [26]. (**b**) Underside of a Western Painted Turtle. Photo by Matt Young [27].

This intensely colorful turtle (Figure 2) has existed for approximately 15 million years, according to the fossil record [25]. The genomes of three turtles have recently been sequenced, including the green sea turtle (*Chelonia mydas*) and the Chinese softshell turtle *Pelodiscus sinensis* [28], as well as the western painted turtle, *Chrysemys picta bellii* [29,30]. Interestingly, these papers place the turtles as a sister group to crocodiles and birds, which is different than their prior morphological classification [31]. The precise evolutionary relationship of the testudine class with other reptiles is a matter of current debate [32,33,34,35,36]. Their genomes appear to encode cathelicidin antimicrobial peptides that are “snake-like” but encode defensin type peptides (gallinacin-like) that may be more similar to “avian” antimicrobial peptides.

### 2.2. Sphenodontia (Tuataras)

**Figure 3:**
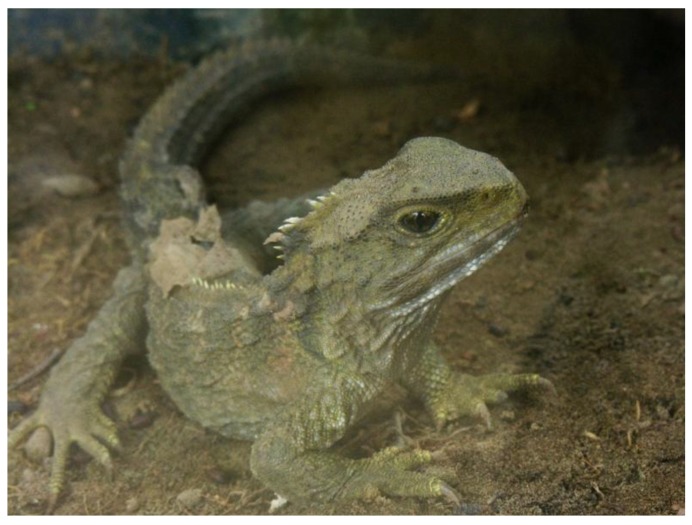
*Sphenodon punctatus*, Tuatara, Nga Manu, Waikanae, New Zealand. Photo by PhillipC [39].

The family Sphenodontidae (within Lepidosauromorpha) contains the genus of Sphenodon, which has one species, *S. puctatus* (previously thought to have three species including *S.* *guntheri*, and *S. diversum*, now considered sub-species) all commonly referred to as tuatara lizards [37,38], although they are not classified as lizards. The tuatara (Figure 3) is the farthest removed from the avian lineage in terms of evolution and is considered to be the most “ancient” extant reptile, in existence in its current form for 100 million years.

The tuatara lives only on the costal islands of New Zealand [40]. There are currently significant efforts underway to sequence the tuatara genome [41]. However, until the genomes and transcriptomes can be analyzed for antimicrobial peptides and verified from tuatara samples, there are no known antimicrobial peptides from the tuatara.

### 2.3. Squamata (Snakes and Lizards)

**Figure 4:**
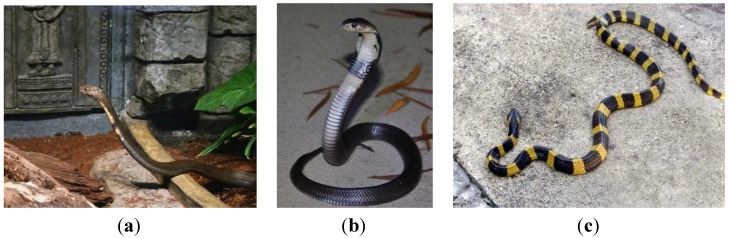
Elapid snakes (**a**) The King cobra (*O. hannah*) [44] (**b**) A juvenile Chinese cobra *(N. atra)* [45] (**c**) Banded Krait *(B. fasciatus*) [46].

The Squamata order of Lepidosauromorpha includes lizards and snakes. In the group of snakes which although limbless are considered tetrapod vertebrates and are descended from four legged ancestors, we will consider elapid snakes and pythons. Elapid snakes are venomous, fanged snakes commonly found in warm climates. Pythons are nonvenomous snakes that are found in Africa, Asia and Australia. The King cobra (*Ophiophagus (O.) hannah*) is one of the longest venomous snakes [42], with lengths up to 18 ft (Figure 4a). The genus *Naja* is a group of venomous elapid snakes in southern Africa and South Asia, including the species *Naja (N.) atra*, the Chinese King cobra (Figure 4b). The Banded Krait (*Bungarus (B.) fasciatus*) is commonly found in Southeast Asia and India and has distinctive banding markings (Figure 4c). The Burmese python (*Python bivittatus*) is typically found in tropical areas including Southeast Asia and is one of the largest snakes, typically reaching 12 ft long. This snake is also found as an invasive species in the Florida Everglades in the Southeast United States [43]. Snakes encode well-studied cathelicidin peptides that appear to be generally similar throughout the reptiles, despite the well-known sequence diversity of cathelicidin antimicrobial peptides in general.

**Figure 5:**
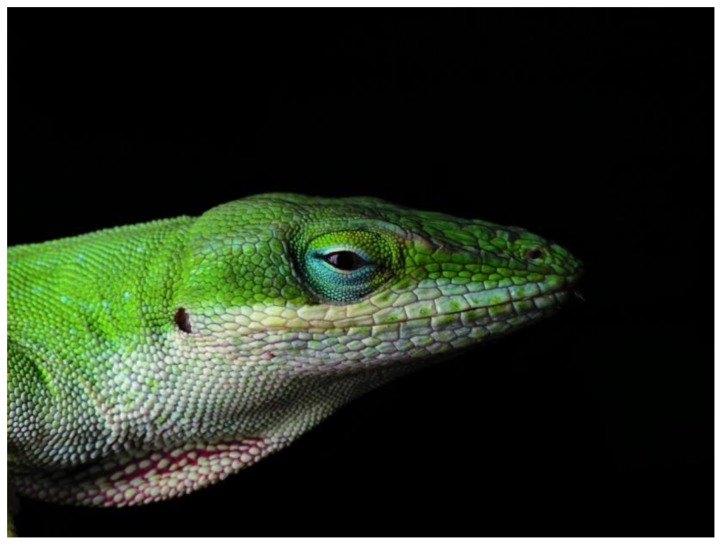
Male Carolina Anole with partially expanded dewlap [47].

The Squamata order also includes the lizards. Unlike snakes, lizards have four legs and external ears. There are more than 6,000 species of lizards, making it the largest group of reptiles. Lizards inhabit a wide range of ecological niches, from hot, dry desserts to cool, moist forests. The main groups of lizards include Gekkota, Iguania, Scincomorpha and Platynota (Varanoidea). Although the tuatara looks much like a lizard, it is excluded from the lizard group. The Gekkota suborder includes geckos, while skinks fall into the suborder Scincomorpha. Monitor lizards, Komodo dragons and Gila monsters are classified as Platynota (Varanoidea). The suborder Iguania includes chameleons, anoles and iguanas. The Carolina green anole lizard (*Anolis carolinensis)* is a small tree-living lizard, often green or brown, commonly found in the southeastern United States. It has intense green, blue and white coloring, especially evident on the male, and has a pink dewlap on its throat (Figure 5). The Anole lizard genome encodes for many β-defensin antimicrobial peptide genes and lysozyme genes.

The genomes of several members of the Squamata have recently been sequenced including the anole lizard [23], the elapid snakes *O. hannah* [15], *B. fasciatus*, and *N. atra* [30]. High-throughput sequencing and mass-spectrometry proteomics has been recently performed on snake venom, which should provide a deep view of proteins and peptides produced in the venom [48]. Antimicrobial peptides have been identified in the anole (defensins) [49], as well as cathelicidins in all three species of elapid snakes (see below) [3]. Very recently, the python genome has been sequenced [30]. Genome projects are also underway for other reptiles, such as the common garter snake (*Thamnophis sirtalis*) [23].

### 2.4. Crocodilia (Crocodilians)

**Figure 6:**
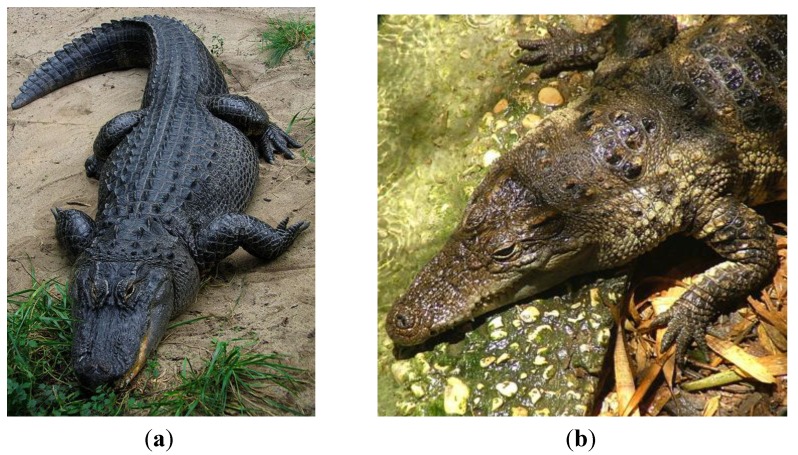
Crocodilians. (**a**) The American alligator, *Alligator mississippiensis* [52]. (**b**) The Siamese crocodile, *Crocodylus siamesnsis* [53].

The order of crocodilians (Crocodilia) within the group Archosauromorpha includes alligators and caimans (family Alligatoridae), crocodiles (family Crocodylidae), and gharials (gavials, family Gavialidae) (Figure 6). Most species live in fresh-water, but a few have also adapted to salt-water conditions. These animals are evolutionarily ancient, and along with birds, are considered the only surviving relatives of dinosaurs (Archosauria). Within their semi-aquatic ecosystems, these large animals are the apex predators, using their powerful jaws to capture large and small prey [50]. They are cold-blooded and egg-laying. They can be found around the world, in the Americas, in Asia, South America, and Africa [51]. The ancestors of alligators and crocodiles first emerged in the Late Cretaceous era, and the modern alligators and crocodiles are estimated to be as much as 83 million years old.

Today, the American alligator is commonly found all around the Gulf of Mexico and Southeastern United States, having made a comeback from near extinction due to overhunting. The meat and skins of farmed alligators are now harvested for consumption and use. This large animal is typically 8–11 ft long and 200–500 lbs depending on gender and age [54]. The alligator has a broad snout and the top teeth come down over the bottom lips, unlike the crocodile. Alligators inhabit swamps such as the Great Dismal Swamp, rivers and streams, ponds and lakes, with a preference for fresh water over brackish water, and intolerance for salt water. Alligators can be infected by *Mycoplasma alligatoris* [55] as well as other bacterial pathogens. In terms of temperature preference, alligators are more cold tolerant than most other crocodilian species, accounting for their location as far north as South Carolina in the United States [54].

The Siamese crocodile (*Crocodylus siamensis*) [56,57] is a critically endangered freshwater crocodilian found in Southeast Asia (Cambodia, Vietnam, Thailand, Indonesia and Burma for example). Like most crocodiles, it is a tropical animal with low tolerance for the cold [3]. This animal is smaller than the alligator, typically about 7 ft long and 80–150 lbs when fully grown, although larger specimens have been recorded [4]. These animals are being intensively studied as part of their conservation, and are bred in captivity, and thus are accessible to researchers for DNA or blood samples [58,59,60].

Crocodilians such as alligators can carry a high burden of fecal coliforms from their aquatic environment [61], yet despite their potentially near-constant exposure to potential pathogens, alligators and other crocodilians do not seem to be susceptible to infection by these organisms, either systemically or on their skin, via wounds or lesions. This has led to the idea that these animals may have highly potent antimicrobial components to their immune systems [57,62,63,64,65,66,67]. Several crocodilian genomes have been published [68] or are underway [69]. The peptides that have been discovered in crocodilians are outlined in the sections below and include lysozyme, defensin, hepcidin, hemocidin and other antimicrobial peptides. Several important crocodilian genomes have been sequenced, including the Chinese alligator (*Alligator sinensis*), the American alligator (*Alligator mississippiensis*), the saltwater crocodile (*Crocodylus porosus*) and the Indian gharial (*Gavialis gangeticus*) [69,70]. It will be very interesting to compare freshwater species to saltwater species in terms of their innate immune response profiles, as the environmental organisms will be significantly different between the two types of water.

## 3. Antimicrobial Peptides of Reptiles

Reptiles are highly adapted to their environments, which often include many bacteria, allowing them to be such successful animals. Antimicrobial peptides are part of the innate immune system, and may contribute to the survival of reptiles. However, to date there has been no direct data in reptiles such as gene knock-out experiments to demonstrate their importance to overall survival, development and resistance to infection, as has been done in mice [71]. There has been some suggestion that β-defensin peptides are associated with fur color in dogs [72,73], but again no studies of this kind have yet been done on reptiles. This area of research presents many opportunities for scientists to explore the role of antimicrobial peptides in these animals. This paper will review the known (Table 1) and predicted antimicrobial peptides of reptiles. Recently, new reptilian genomes have been sequenced. From these genomes, additional antimicrobial peptide genes have been predicted here by bioinformatics analysis (Table 2, Table 3, Table 4, Table 5, Table 6, Table 7 and Table 8), which should be synthesized and tested for activity.

Antimicrobial peptides are well-characterized peptides, and exhibit significant structure-function specificity. Following the identification of magainin peptides in amphibians in 1987 [74], scientists have been exploring the diversity of peptides expressed in different animals, looking for new structures and new functions of antimicrobial peptides. The structure and function of each of the major classes of antimicrobial peptides is described below, in addition to the data pertaining to its known or predicted expression in reptiles.

### 3.1. Defensin Peptides in Reptiles

Defensins are one of the major classes of antimicrobial peptides in higher vertebrates. These 3–4 kDa cationic peptides are characterized by having six cysteines arranged in three disulfide bonds, with the characteristic pairing of the bonds highly characteristic for each type of defensin. Defensins have predominantly β-sheet characteristic with some α-helices. Defensins are encoded in the genome and are processed from a pro-defensin molecule by proteases [75].

#### 3.1.1. Three Sub-Classes of Defensins

Defensins are known to be critical components of innate immunity in many animals [76]. The three main sub-classes of defensins are α, β- and θ-defensins. In humans, α-defensins are commonly found in neutrophils and other leukocytes (for example, Human Neutrophil peptide HNP-1 = α-defensin 1), and are important in the ability of white blood cells to deal with pathogens [77]. The β-defensins are defined as having a Cys1-Cys5, Cys2-Cys4, and Cys3-Cys6 bonding pattern of cysteines. In humans, β-defensins are commonly expressed in epithelial cells, and are widely expressed in the body [78]. The expression of these cationic antimicrobial peptides are often induced following bacterial or viral infection as part of the innate immune response, except for hBD1 which appears to be constitutively expressed in humans. The third class of defensins is the θ-defensins. θ-Defensins are not known outside of primates, and are not expressed in humans [21] but are very active antiviral peptides [76].

Within the reptiles, there are no known genes for α-defensins, and only the β-defensins appear to be expressed, similar to what is found in avians. Within some species of reptiles, β-defensin genes and peptides have been identified, for example in the red-eared slider turtle [79]. Lizards are known to be highly resistant to infection, and recently genes encoding up to 32 different β-defensin-like peptides were identified in the *Anole carolinensis* genome [49]. Indeed, using an antibody that reacts to AcBD15 (one of those β-defensin-like peptides in anole), staining was observed in some (but not all) of the granules of heterophilic and basophilic granulocytes in lizards, a snake, a turtle and the tuatara, but not alligator nor chicken granulocytes. Cells from other tissues such as epidermis were negative for staining. Alibardi *et al.* describe that not all the granules within a granulocyte stained with the antibody, suggesting that there may be different types of granules as is seen in mammalian neutrophils [22].

Similarly, turtle leukocytes were found to contain TBD-1, the first β-defensin identified in reptile leukocytes [14]. In the turtle system, TBD-1 was also identified in other tissues, such as the skin [18,80,81]. These fascinating and unique results suggest that further study of the antimicrobial peptidome of reptilian granulocytes should be performed.

#### 3.1.2. Inducible Expression of β-Defensins in Wounded Lizards

The first extensive report of an *in vivo* role for β-defensin peptide expression was in the anole lizard (Table 2). It has long been known that lizards can lose their tails as a method of predator escape, and that these tail then regenerate from the wound site. In this process, a wound is formed, which does not typically get infected. β-Defensin peptides are found to be expressed both within the azurophilic granulocytes in the wound-bed as well as in the associated epithelium [82,83], and are observed in phagosomes containing degraded bacteria. While there is a distinct lack of inflammation in the wound, which is associated with regeneration, there is a high level of expression of AcBD15 and AcBD27 (two of the most highly expressed β-defensins in that tissue) [84,85]. Overall, there appears to be a fascinating role of AcBD15 and AcBD27 in the wound healing and regeneration in the anole lizard.

**Table 2:** Predicted defensin-like protein genes in multiple reptilian species.

Organism	Peptide annotation	aa	Locus- Accession #
*Alligator mississippiensis*	Gallinacin-14-like	58	XP_006270781.1
*Alligator sinensis*	Gallinacin-14-like	58	XP_006033878.1
*Anolis carolinensis*	β-Defensin-like protein 5	62	CBY85058.1
*Anolis carolinensis*	β-Defensin-like protein 8	65	CBY85059.1
*Anolis carolinensis*	β-Defensin-like protein 9	66	CBY85060.1
*Anolis carolinensis*	β-Defensin-like protein 10	67	CBY85061.1
*Anolis carolinensis*	β-Defensin-like protein 15	63	CCA62931.1
*Anolis carolinensis*	β-Defensin-like protein 21	89	CBY85062.1
*Anolis carolinensis*	β-Defensin-like protein 22	95	CBY85063.1
*Anolis carolinensis*	β-Defensin-like protein 27	81	CBY85064.1
*Anolis carolinensis*	Gallinacin-10-like	68	XP_003225602.1
*Anolis carolinensis*	Gallinacin-13-like	60	XP_003225598.1
*Anolis carolinensis*	Hypothetical protein LOC100555370		XP_003227809.1
*Anolis carolinensis*	Hypothetical protein LOC100555565		XP_003227810.1
*Anolis carolinensis*	Hypothetical protein LOC100555756		XP_003227811.1
*Anolis carolinensis*	Hypothetical protein LOC100562305		XP_003225604.1
*Anolis carolinensis*	Hypothetical protein LOC100562502	65	XP_003225605.1
*Anolis carolinensis*	Hypothetical protein LOC100562898		XP_003225607.1
*Anolis carolinensis*	Hypothetical protein LOC100563098		XP_003225608.1
*Chrysemys picta bellii*	β-Defensin 1-like	80	XP_005308390.1
*Chrysemys picta bellii*	Gallinacin-5-like		XP_005290738.1
*Chrysemys picta bellii*	Gallinacin-5-like, partial		XP_005314963.1
*Chrysemys picta bellii*	Gallinacin-14-like	58	XP_005308403.1
*Pelodiscus sinensis*	Gallinacin-1 α-like		XP_006137072.1
*Pelodiscus sinensis*	Lingual antimicrobial peptide-like isoform X2		XP_006127561.1
*Bothrops neuwiedi*	β-Defensin-like protein		AGF25392.1
*Bothrops jararacussu*	β-Defensin-like protein		AGF25388.1
*Bothrops leucurus*	β-Defensin-like protein		AGF25389.1
*Bothrops matogrossensis*	β-Defensin-like protein		AGF25391.1, AGF25390.1
*Bothrops diporus*	β-Defensin-like protein		AGF25384.1
*Bothrops pauloensis*	β-Defensin-like protein		AGF25393.1
*Bothrops jararaca*	β-Defensin-like protein		AGF25386.1, AGF25387.1
*Bothrops atrox*	β-Defensin-like protein		AGF25383.1
*Bothrops erythromelas*	β-defensin-like protein		AGF25385.1

#### 3.1.3. Expression of β-Defensins in Reptile Eggs

Reptile eggs are a good biological sample for the purification of peptides and proteins, since there is so much material within each egg. Recently, it was found that while the β-defensin-like peptide pelovaterin (Table 1) identified in the eggshell of the Chinese soft-shelled turtle does indeed have antimicrobial activity, these peptides may also play an additional role in the formation of the eggshell, through aggregation [15]. This is similar to the role of the gallin defensin-like peptide in avian eggs [86,87,88,89,90].

### 3.2. Cathelicidin Peptides in Reptiles

Cathelicidins are a second major class of antimicrobial peptides in higher eukaryotes. They are characterized as being antimicrobial peptides derived by proteolytic cleavage from a pre-propeptide that includes the cathelin domain in mammals and less-well conserved cathelin domain in other eukaryotes [91,92]. In humans, cathelicidins are stored in the azurophilic granules of neutrophils as the inactive prepropeptide, and are processed by enzymes (neutrophil elastase [93] or a serine protease [19]) to the mature active peptide [94]. In humans and higher vertebrates, the active cathelicidin peptide is always encoded on Exon 4 of the cathelicidin encoding gene [91,95,96]. Four cathelicidin-like peptides have been identified in the chicken (*Gallus gallus domesticus*) [97], including fowlicidin-1, -2 and -3 (also known as chCATH-1, chCATH-2/CMAP27, chCATH-3) [98], and chCATH-B1/chCATH-4 [99]. In humans, the hCAP18 cathelicidin is processed by proteinase 3 inside the granule [19] or neutrophil elastase in the extracellular space [4] to its active form. In the case of chicken cathelicidin, the pre-pro-cathelin domain peptide is cleaved by a serine protease to release the mature peptide following stimulation of heterophils with LPS [100].

### 3.3. Cathelicidin Peptides in Snakes

Cathelicidin peptides have been identified and characterized in the snake family (Table 1) [1,2], although there may be similar genes in other reptiles by genomic analysis (see below). Highly related cathelicidins were identified in *B. fasciatus*, *O. hannah* and *N. atra* [3] (Table 3). We identified additional genes for antimicrobial peptides by BLAST searching the genomes of the pit vipers (*Bothrops atrox*, *Trimeresurus wagler* and *Crotalus durissus*) [13] and the Eastern brown snake (*Pseudonaja textilis*) (Table 3). The genes of these cathelicidins have the general structure of the cathelicidin genes, including a poorly conserved cathelin domain and the c-terminal active peptide. The snake cathelicidins have been well studied. The functional cathelicidin peptide of the snake family is highly divergent from the functional cathelicidin peptide of humans, for example, confirming the “general rule” of cathelicidins, which is that the active peptides are highly divergent, but the pre-pro-regions in the gene and the peptide are more highly conserved [101].

#### 3.3.1. King Cobra (*Ophiophagus (O.) Hannah*)

Zhao *et al.* determined the hemolytic and antimicrobial activity of the predicted *O. hannah* cathelicidin, OH-CATH [3] (Table 1). The OH-CATH peptide proved to be an excellent inhibitor of bacterial growth and demonstrated broad-spectrum activity against bacterial isolates including multi-drug resistant strains. The OH-CATH peptide displayed greater potency than the human cathelicidin LL-37 against a variety of known human bacterial pathogens and the antimicrobial potency of OH-CATH was not significantly impacted by the concentration of salt in the media. They demonstrated that OH-CATH showed no hemolytic activity against erythrocytes, even at a concentration of 200 μg/mL, suggesting low cytotoxicity of the peptide to eukaryotic cells [3]. Additional studies with smaller fragments of OH-CATH (Table 1) have also been tested and found to be active both *in vitro* and *in vivo,* even against antibiotic resistant bacteria [4,6]. Their strong antimicrobial activity and lack of hemolytic activity make the reptile cathelicidins strong candidates for development into new therapeutics.

**Table 3:** Known and predicted cathelicidin open reading frames in multiple snake species. The active antimicrobial peptides NA-CATH, BF-CATH and OH-CATH are highlighted.

Protein name [Organism]	Accession	Sequence	
Cathelicidin-NA antimicrobial peptide [Naja atra]	B6S2X0.1	1	MEGFFWKTLLVVGALTISGTSSFPHKPLTYEEAVDLAVSVYNSKSG EDSLYRLLEAVPALKWDALSESNQELNFSVKETV 80
		81	CQMAEERSLEECDFQEAGAVMGCTGYYFFGESPPVLVLTCKSVGNE- EEQKQEEGNEEEKEVEKEEKEEDQKDQPKR 156
		157	V 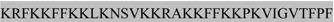 191
Cathelicidin-BF antimicrobial peptide [Bungarus fasciatus]	B6D434.1	1	MEGFFWKTLLVVGALAIAGTSSLPHKPLIYEEAVDLAVSIYNSKSGEDS LYRLLEAVSPPKWDPLSESNQELNFTMKETV 80
		81	CLVAEERSLEECDFQEDGAIMGCTGYYFFGESPPVLVLTCKPVGEE- EEQKQEEGNEEEKEVEKEEKEEDEKDQPRR 156
		157	V 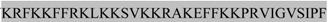 191
Cathelicidin-OH antimicrobial peptide [Ophiophagus hannah]	B6S2X2.1	1	MEGFFWKTLLVVGALAIGGTSSLPHKPLTYEEAVDLAVSIYNSKSGEDSL YRLLEAVPPPEWDPLSESNQELNFTIKETV 80
		81	CLVAEERSLEECDFQEDGVVMGCTGYYFFGESPPVVVLTCKPVGEE- GEQKQEEGNEEEKEVEEEEQEEDEKDQPRR 156
		157	V 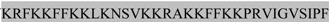 191
cathelicidin-like peptide precursor [Bothrops atrox]	AGS36140.1	1	MQGFFWKTWLVVALC--GTSSSLAHRPLSYGEALELALSIYNSKAG EESLFRLLEAVPQPEWDPLSEGSQQLNFTIKETV 78
		79	CQVEEERPLEECGFQEDGVVLECTGYYFFGETPPVVVLTCVPVGG V-EEEEEDE-EEQKAEVEKDEKEDEEKDRPKR 154
		155	VKRFKKFFKKLKNSVKKRVKKFFRKPRVIGVTFPF 189
cathelicidin-related antimicrobial peptide isoform precursor [Pseudonaja textilis]	AGS36144.1	1	MEGFFWKTWLVVAAFAIGGTSSLPHKPLTYEEAVDLAVSTYNGKSGEE SLYRLLEAVPPPKWDPLSESNQELNLTIKETV 80
		81	CLVAEERSLEECDFQDDGAVMGCTGYFFFGESPPVLVLTCEPLGED-EE QNQEEE-------EEEEKEEDEKDQPRR 149
		150	VKRFKKFFMKLKKSVKKRVMKFFKKPMVIGVTFPF 184
cathelicidin-related antimicrobial peptide precursor [Pseudonaja textilis]	AGS36143.1	1	MDGFFWKTWLVVAALAIGGTSSLPHKPLTYEEAVDLAVSTYNGKSGEES LYRLLEAVPPPKWDPLSESNQELNLTIKETV 80
		81	CLVAEERSLEECDFQDDGAVMGCTGYFFFGESPPVLVLTCEPLGED- EEQNQEEE-------EEEEKEEDEKDQPRR 149
		150	VKRFKKFFRKLKKSVKKRVKKFFKKPRVIGVTIPF 184
cathelicidin-like peptide precursor [Bothrops lutzi]	AGS36141.1	1	MQGFFWKTLLVVALC—GTSSSLAHRPLSYGEALELALSVYNSKAGE ESLFRLLEAVPQPEWDPLSEGSQQLNFTIKETV 78
		79	CQVEEERPLEECGFQEDGVVLECTGYYFFGETPPVVVLTCVPVGG V-EEEEEDE-EEQKAEVEKDEKEDEEKDRPKR 154
		155	VKRFKKFFKKLKNNVKKRVKKFFRKPRVIGVTIPF 189
cathelicidin-like peptide precursor [Lachesis muta rhombeata]	AGS36142.1	1	MQGFFWKTWLVLAVC--GTPASLAHRPLSYGEALELAVSVYNGKAGEAS LYRLLEAVPQPEWDPSSEGSQQLNFTLKETA 78
		79	CQVEEERSLEECGFQEDGVVLECTGYYFFGETPPVVVLSCVPVGGV eEEEEEEE-EEQKAEAENDEKEDEEKDQPKR 159
		160	160 VKRFKKFFKKVKKSVKKRLKKIFKKPMVIGVTFPF 194
cathelicidin-like peptide precursor [Crotalus durissus terrificus]	AGS36138.1	1	MQGFFWKTWLVLAVC—GTPASLAHRPLSYGEALELAVSVYNGKAG EASLYRLLEAVPQPEWDPSSEGSQQLNFTLKETA 78
		79	CQVEEERSLEECGFQEDGVVLECTGYYFFGETPPVVVLSCVPVGGV eEEEEEEE-EEQKAEAENDEKGDEEKDQPKR 159
		160	VKRFKKFFKKVKKSVKKRLKKIFKKPMVIGVTIPF 194

#### 3.3.2. Chinese King Cobra *(Naja (N.) Atra)*

The genus *Naja* is a group of venomous elapid snakes in the southern Africa and South Asia, including the species *N. atra*, the Chinese King cobra. The cathelicidin peptide from the Chinese King cobra, *N. atra* (NA-CATH) (Table 1), has been well studied. The full-length NA-CATH peptide was synthesized and was found to be antimicrobial against a wide variety of bacteria [102,103,104,105]. Smaller fragments of this peptide have been identified and found to be effective [102,103,104,105].

Interestingly, while the human cathelicidin peptide (LL-37) was found to exert antibiofilm activity against bacterial pathogens such as *Pseudomonas* [103] and *Staphylococcus* [105], NA-CATH did not exhibit antibiofilm effect against *Pseudomonas*, despite similar overall biophysical properties (length, size, charge) to LL-37.

**Figure 7:**
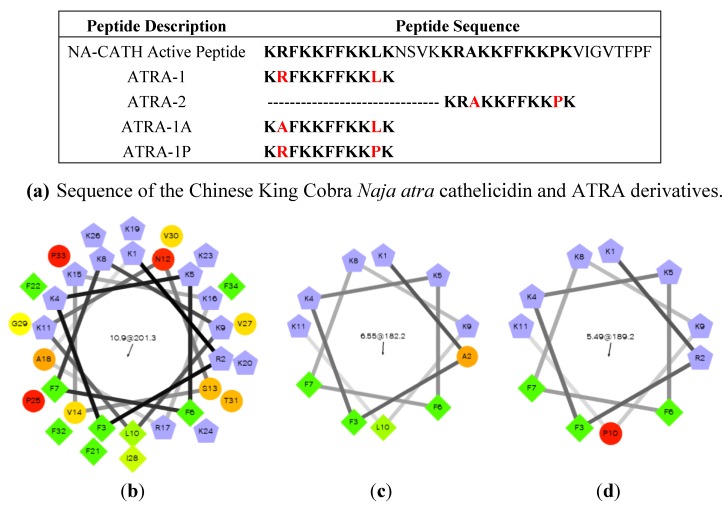
*Naja atra* cathelicidin peptide analysis. (**a**) Sequences of the NA-CATH active peptide and derivatives [102,103,104,105]. (**b**) Helical wheel projection of NA-CATH. (**c**) Analysis of the active ATRA-1A peptide (**d**) Analysis of the inactive ATRA-1P peptide. From Rzlab.ucr.edu/scripts/wheel/wheel.cgi: “The hydrophilic residues are presented as circles, hydrophobic residues as diamonds, potentially negatively charged as triangles, and potentially positively charged as pentagons. Hydrophobicity is color coded as well: the most hydrophobic residue is green, and the amount of green is decreasing proportionally to the hydrophobicity, with zero hydrophobicitycoded as yellow. Hydrophilic residues are coded red with pure red being the most hydrophilic (uncharged) residue, and the amount of red decreasing proportionally to the hydrophilicity. The potentially charged residues are light blue.”

The smallest identified fragment of NA-CATH is an 11-amino acid peptide, called ATRA, which imperfectly repeats in the NA-CATH sequence (Figure 7a) [102]. Variants of the ATRA peptide have been found to be very informative regarding the role of charged residues and proline residues in the activity of small antimicrobial peptides [102]. The peptide ATRA-1 is almost identical to the ATRA-2 peptide, with the exception of the 3d (A/F) and 10th (L/P) residues. Variants of these peptides were made to switch out the 3rd and 10th position amino acids, such that ATRA-1A contains alanine at position 3 rather than phenylalanine (F). Another variant was ATRA-1P, which contains proline at position 10 rather than leucine (L). All the peptides were the same length, similar molecular weight, and the same net charge. It was found for multiple gram-negative bacteria that ATRA-1P was almost as ineffective as ATRA-2, while ATRA-1A was as effective as ATRA-1 [102]. These results suggested that the introduction of a proline in ATRA-1P may have affected the charge distribution on that peptide, which then may disrupt the antibacterial activity of the peptide (Figure 7b,c).

Further studies have examined the d-amino acid enantiomer of the ATRA peptides [106], and found that d-ATRA-1A is as effective as ATRA-1A, and as it is protease resistant, potentially has a longer half-life. The ATRA peptides have also been demonstrated to be highly antimicrobial both *in vitro* and *in vivo* against both gram-negative bacteria such as *Escherichia coli* K12 strain and *Aggregatibacter actinomycetemcomitans-*Y4 [102], *Pseudomonas aeruginosa* [103] and *Francisella novicida* [104]. ATRA peptides are also antimicrobial against the gram-positive bacteria *Staphylococcus aureus* [105].

#### 3.3.3. Banded Krait *(B. fasciatus)*

The cathelicidin peptide from *B. fasciatus* (BF-CATH, Table 1, Table 3) is also very well studied. The full-length BF-CATH peptide has been shown to have antimicrobial activity against a variety of bacteria including antibiotic resistant bacteria [7,107]. Smaller fragments of BF-CATH have also been expressed and studied [10,108] (Table 1). For example, BF-30 showed significant activity against drug-resistant *E. coli* and *S. aureus* both *in vitro* and *in vivo* [109]. BF-15 is a shorter version of BF-CATH with lower hemolytic activity, and broad-spectrum antimicrobial activity even against antibiotic-resistant bacteria [8].

#### 3.3.4. Predicted Cathelicidins in Other Snake Species

By performing BLAST analysis with the elapid snake cathelicidins against other snake sequences in the database, we identified cathelicidin-like gene sequences in the venomous pit viper, *Bothrops atrox*, and the Eastern brown snake, *Pseudonaja textilis* (Table 3). These genes should be further studied to verify that cathelicidin-like peptides are produced and to determine the activity of these peptides.

### 3.4. Cathelicidin Peptides in Lizards

Cathelicidin-like peptides Ac-CATH-1, Ac-CATH-2a, Ac-CATH -2b, and Ac-CATH -3 have been reported in the genome of the Carolina anole lizard (*Anolis carolinensis*) [110] (Table 4). In addition, cathelicidin 1 and 2 antibody reactive peptides have been identified by immunocytochemistry staining within granules of heterophilic and basophilic granulocytes [111]. This study also identified cathelicidin-antibody staining material in wound epidermis and associated with bacteria within wounds [111].

**Table 4:** Predicted active cathelicidin peptides in the anole lizard [91,92].

Organism	Peptide annotation
*Anolis carolinensis*	Ac-CATH-1 MGRITRSRWGRFWRGAKRFVKKHGVSIALAGLRFG (+10)
*Anolis carolinensis*	Ac-CATH-2a/b DPQMTRFRGLGHFFKGFGRGFIWGLNH (+3)
*Anolis carolinensis*	Ac-CATH-3—no active peptide encoded.

### 3.5. Cathelicidin Peptides in Turtles

Analysis of the recently published genomes revealed that there are many cathelicin-like genes in turtles. While no active cathelicidin peptides have been demonstrated yet in the turtle (Table 1), there are at many predicted cathelicidin-like peptide genes (Table 5). Many of these are annotated as being similar to the snake cathelicidins (eg OH-CATH).

**Table 5:** Predicted cathelicidin pre-pro-protein genes in multiple turtle species.

Organism	Peptide annotation	Locus- Accession #
*Chrysemys picta bellii*	Cathelicidin-OH antimicrobial peptide-like	XM_005295113.1
*Chrysemys picta bellii*	Cathelicidin-OH antimicrobial peptide-like	XP_005295170.1
*Chrysemys picta bellii*	Cathelicidin-2-like	XP_005295171.1
*Chrysemys picta bellii*	Uncharacterized LOC101951069	XR_255838.1
*Chrysemys picta bellii*	Uncharacterized LOC101951243	XR_255839.1
*Pelodiscus sinensis*	Cathelicidin-2-like	XM_006114422.1
*Pelodiscus sinensis*	Cathelicidin-2-like	XM_006114419.1
*Pelodiscus sinensis*	Cathelicidin-OH antimicrobial peptide-like transcript variant X1	XM_006129620.1
*Pelodiscus sinensis*	Cathelicidin-OH antimicrobial peptide-like transcript variant X2	XM_006129621.1
*Pelodiscus sinensis*	Cathelicidin-OH antimicrobial peptide-like	XM_006129625.1
*Pelodiscus sinensis*	Cathelicidin-BF antimicrobial peptide-like	XP_006114480.1
*Pelodiscus sinensis*	Cathelicidin-BF antimicrobial peptide-like	XM_006114418.1
*Pelodiscus sinensi*	Cathelicidin-2-like	XP_006114484.
*Pelodiscus sinensis*	Cathelicidin-2-like	XP_006114481.1
*Pelodiscus sinensis*	Cathelicidin-OH antimicrobial peptide-like isoform X1	XP_006129682.1
*Pelodiscus sinensis*	Cathelicidin-OH antimicrobial peptide-like isoform X2	XP_006129683.1
*Pelodiscus sinensis*	Cathelicidin-OH antimicrobial peptide-like	XP_006129687.1
*Chelonia mydas*	Hypothetical protein UY3_13361	EMP29519.1
*Chelonia mydas*	Hypothetical protein UY3_13360	EMP29518.1

### 3.6. Cathelicidin Peptides in Crocodilians

Analysis of the databases revealed that there are many cathelicidin-like genes in alligators. While no active cathelicidin peptides have been demonstrated yet in the alligator (Table 1), there are at many predicted cathelicidin-like peptide genes (Table 6). Many of these genes are annotated as being similar to the snake cathelicidins (e.g., OH-CATH).

**Table 6:** Predicted cathelicidin pre-pro-protein genes in Alligator species.

Organism	Peptide annotation	Locus- Accession #
*Alligator mississippiensis*	Cathelicidin-2-like	XM_006262429.1
*Alligator mississippiensis*	Cathelicidin-2-like	XP_006262491.1
*Alligator mississippiensis*	Cathelicidin-OH antimicrobial peptide-like	XM_006262431.1
*Alligator mississippiensis*	Cathelicidin-OH antimicrobial peptide-like	XM_006262430.1
*Alligator mississippiensis*	Cathelicidin-OH antimicrobial peptide-like	XP_006262492.1
*Alligator mississippiensis*	Cathelicidin-OH antimicrobial peptide-like	XP_006262493.1
*Alligator sinensis*	Cathelicidin-2-like	XM_006026412.1
*Alligator sinensis*	Cathelicidin-2-like	XP_006026474.1
*Alligator sinensis*	Cathelicidin-2-like	XP_006026475.1
*Alligator sinensis*	Cathelicidin-3-like	XM_006026409.1
*Alligator sinensis*	Cathelicidin-3-like	XP_006026471.1
*Alligator sinensis*	Cathelicidin-OH antimicrobial peptide-like	XM_006037224.1
*Alligator sinensis*	Cathelicidin-OH antimicrobial peptide-like	XM_006026410.1
*Alligator sinensis*	Cathelicidin-OH antimicrobial peptide-like	XM_006026411.1
*Alligator sinensis*	Cathelicidin-OH antimicrobial peptide-like	XM_006037211.1
*Alligator sinensis*	Cathelicidin-OH antimicrobial peptide-like	XP_006037286.1
*Alligator sinensis*	Cathelicidin-OH antimicrobial peptide-like	XP_006026472.1
*Alligator sinensis*	Cathelicidin-OH antimicrobial peptide-like	XP_006037273.1
*Alligator sinensis*	Cathelicidin-OH antimicrobial peptide-like	XP_006026487.1

### 4.1. Liver-Derived Peptides in Reptiles

#### 4.1.1. Hepcidin (HAMP1)

Hepcidin antimicrobial peptides are liver-expressed peptides containing eight cysteines (four disulfide bonds) that are broadly antimicrobial, binds iron and is involved in ferroportin binding [112,113]. A gene encoding a hepcidin-like peptide (E8ZAD0_CROSI) was recently proposed to be encoded by the Siamese crocodile (*Crocodylus siamensis*). A 26 aa peptide (Cshepc), representing the predicted active peptide (FNSHFPICSYCCNCCRNKGCGLCCRT), was expressed in yeast, and the unpurified product was found to have antimicrobial activity against both Gram-positive bacteria such as *S. aureus* and *Bacillus subtilis*, as well as Gram-negative bacteria *Escherichia coli* and *Aeromonas* *sobria* [114]. Hepcidin-like sequences were also identified in the anole lizard, although interestingly hepcidins appear to be missing in most avians [115].

#### 4.1.2. LEAP-2, Liver Expressed Antimicrobial Peptide-2

Another liver-expressed peptide, LEAP-2, is expressed in many different organisms, has broad-spectrum antibacterial and antifungal activity, and can be induced following bacterial challenge [116,117,118]. This 40 aa, 4-cysteine, cationic peptide differs significantly from hepcidin in sequence and predicted structure [119], but is also predominantly expressed in the liver.

The Leap-2 gene is annotated in many of the sequenced reptiles (Table 7), including *Pelodiscus sinensis*, *Chrysemys picta bellii*, *Alligator sinensis*, *Alligator mississippiensis*, as identified by a BLAST analysis we performed with the LEAP-2 from *Anolis carolinensis*. The antimicrobial activity or biological role of this peptide in reptiles has not been studied.

**Table 7:** Predicted LEAP-2 pre-propeptide amino-acid sequences identified in reptiles.

Species	Predicted LEAP-2 full sequence	Accession Number
*Anolis carolinensis*	MTPLKITAVILICSALLFQTQGASLYPPNSQLVRQR RMTPFWRGISRPIGASCRDNSECSTRLCRSKHCSLRTSQE	XP_003217432.1
*Alligator sinensis*	MHWLKVIAVMLLFALHLFQIHCASLHQPNSQPKRQRRM TPFWRGVSSLRPIGASCRDDIECVTMLCRKSHCSLRTSRE	XP_006023615.1
*Alligator mississippiensis*	MHWLKVIAVMLLFALHLFQIHCASLHQPNSQPKRQRRM TPFWRGVSSLRPIGASCKDDGECITMRCRKSHCSLRTSRE	XP_006263463.1
*Pelodiscus sinensis*	MQCLKVIALLLFCAALLTQTHCASLHHSSSQLTRQRRMTP FWRGISLRPIGALCRHDNECISMLCRKNRCSLRISCE	XP_006128591.1
*Chrysemys picta belli*	MQYLKVIAVLLLCAALLSQIHSASLHRPSSHLTRQRRMTPF WRGISLRPIGAICRDDSECVSRLCRKNHCSIRISRA	XP_005302895.1

### 4.2. Lysozyme in Reptiles

Lysozyme is a very important part of innate immunity in most animals. In humans, it is packaged into neutrophil granules, released in tears and other secretions, and is very effective against a broad spectrum of bacteria. Not surprisingly, reptiles have also been found to use lysozyme as part of their innate immune response. In the crocodilians, a lysozyme-like enzyme was identified with broad-spectrum antibacterial activity [67]. Lysozyme proteins very similar to chicken lysozymes (~130 aa) have been identified from some species of turtles, including the soft shelled turtle (*Trionyx sinensis*), the Asiatic soft shelled turtle (*Amyda cartilagenea*) and the green sea turtle (*Chelonia mydas*) [17,19,120,121,122], although the antimicrobial activity of these molecules has not yet been proven. Lysozyme peptide has also been identified from *Crocodylus siamensis* [67]. Lysozyme-like genes were identified by analysis of reptile genomes (Table 8).

### 4.3. Crotamine Peptides in Reptiles

**Figure 8:**
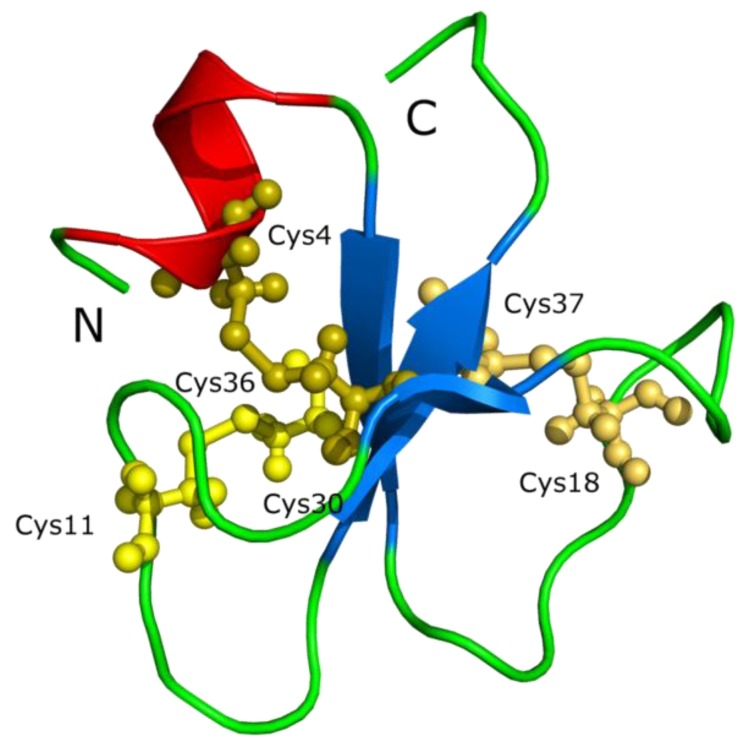
The crotamine chemical structure. Crotamine, a Na+ channel-affecting toxin from *Crotalus durissus terrificus* venom (PDB 1H5O) [125].

Defensins are one example of cysteine-stabilized polypeptides with antimicrobial function. A similar family of peptides is the crotamine toxin family isolated from rattlesnakes with a similar gamma-core motif to defensins [123,124], which is considered a cell-penetrating peptide The sequence of crotamine is YKQ**C**HKKGGH**C**FPKEKI**C**LPPSSDFGKMD**C**RWRWK**CC**KKGSG (+8) [2]. This cationic peptide contains nine lysines (underlined), three disulfide bonds (6 cysteines, shown in bold) and has a defensin-like fold (Figure 8).

**Table 8:** Selected predicted lysozyme genes identified in reptiles.

Reptile name	Enzyme Name	Accession Number
Softshell turtle lysozyme C(SSTL)	Lysozyme C (1,4-β-N-acetylmuramidase C)	Q7LZQ1.3
Asiatic softshell turtle lysozyme C (ASTL)	Lysozyme C	P85345.1
*Pelodiscus sinensis*	Lysozyme	ADR51676.1
*Pelodiscus sinensis*	PREDICTED: lysozyme g-like isoform X2	XP_006113603.1
*Pelodiscus sinensis*	PREDICTED: lysozyme g-like isoform X1	XP_006113602.1
*Pelodiscus sinensis*	PREDICTED: lysozyme g-like	XP_006113601.1
*Chelonia mydas*	Lysozyme C	EMP38935.1
*Chelonia mydas*	Lysozyme G	EMP27176.1
*Chrysemys picta bellii*	PREDICTED: lysozyme C-like	XP_005314893.1
*Chrysemys picta bellii*	PREDICTED: lysozyme C-like	XP_005312037.1
*Chrysemys picta bellii*	PREDICTED: lysozyme g-like protein 2	XP_005283410.1
*Chrysemys picta bellii*	PREDICTED: lysozyme G-like	XP_005283294.1
*Ophiophagus hannah*	Lysozyme C, partial	ETE58503.1
		XP_003225844.1,
*Anolis carolinensis*	Lysozyme C, milk isozyme-like	XP_003216710.1,
		XP_003216704.1
*Anolis carolinensis*	Lysozyme C II-like	XP_003224512.1
*Anolis carolinensis*	Lysozyme g-like	XP_003227178.1
*Alligator sinensis*	Lysozyme C-like	XP_006027022.1, XP_006027021.1
*Alligator sinensis*	Lysozyme G-like	XP_006026406.1, XP_006026397.1, XP_006026395.1, XP_006026396.1

Some reptiles are known to express crotamine-like peptides, and The X-ray structure of crotamine, from the Brazilian snake *Crotalus durissus* *terrificus*, was recently solved (PDB 4GV5_A) [126]. We found that some species of reptiles have genes that are annotated as crotamine-like (Table 9).

**Table 9:** Crotamine-like peptide genes in reptiles.

Reptile	AA	Name	Accession numbers
*Uromastyx aegyptia*	67	crotamine-Uro-1	AGI97143.1
*Crotalus durissus terrificus*	65	crotamine	AAF34911.1, AAF34910.1, AAC02995.1, AAC06241.1
*Crotalus durissus*	34	crotamine	ACA63453.1, ACA63452.1, ACA63451.1, ACA63450.1, ACA63449.1, ACA63448.1, ACA63447.1, ACA63446.1
*Crotalus oreganus helleri*	65	crotamine 7	AEU60015.1
*Crotalus oreganus helleri*	65	crotamine 6	AEU60014.1
*Crotalus oreganus helleri*	65	crotamine 5	AEU60013.1
*Crotalus oreganus helleri*	70	crotamine 4	AEU60012.1
*Crotalus oreganus helleri*	70	crotamine 3	AEU60011.1
*Crotalus oreganus helleri*	83	crotamine 2	AEU60010.1
*Crotalus oreganus helleri*	70	crotamine 1	AEU60009.1
*Pogona barbata*	102	CLP-POGL1	AAZ75614.1
*Pogona barbata*	67	CLP-POGL2	AAZ75615.1
*Pogona barbata*	61	CLP-POGU3	AAZ75613.1
*Pogona barbata*	98	CLP-POGU2	AAZ75612.1
*Pogona barbata*	76	CLP-POGU1	AAZ75611.1
*Varanus tristis*	83	crotamine-Var-5	AGI97148.1
*Varanus glauerti*	83	crotamine-Var-4	AGI97147.1
*Varanus glauerti*	83	crotamine-Var-3	AGI97146.1
*Varanus glauerti*	83	crotamine-Var-2	AGI97145.1
*Varanus glauerti*	83	crotamine-Var-1	AGI97144.1

It has been a matter of some debate whether crotamine peptides are antimicrobial or if they just contain a similar cysteine-stabilized core structure. Recently, it was demonstrated that crotamine toxin is expressed similarly on epithelial or mucosal surfaces, and displays some antimicrobial activity against *Bacillus subtilis*, and was able to permeabilize *Staphylococcus aureus* cells, for example [20]. In addition, crotamine has been shown to be antifungal [127]. Thus, it seems that crotamine should be considered as an antimicrobial peptide of reptiles.

### 4.4. Other Peptides in Reptiles

#### 4.4.1. Leucrocin

There are a handful of peptides identified in reptiles that were not easily classified in the categories above. One interesting example is the peptide leucrocin, an antibacterial compound from white blood cells of the Siamese crocodile (*Crocodylus siamensis*). Unlike Crocosin [65], which does not appear to be peptide based, leucrocins are very small peptides with antibacterial activity. The amino acid sequence of Leucrocin I is NGVQPKY, and of Leucrocin II is NAGSLLSGWG [66]. No known genes encode for these peptides, so their source is unclear. Recently, a synthetic peptide based on the Leucrocin sequence was found to have broad-spectrum antibacterial activity [128].

#### 4.4.2. Omwaprin

Another example of an unusual antimicrobial peptide in reptiles is omwaprin, which is a member of the waprin family of venom proteins (Sequence shown in Table 1) [11,12], and was isolated from the Australian inland taipan (*Oxyuranus microlepidotus*), considered the most venomous snake (a member of the Elapid family of snakes). This 50 aa peptide has relatively salt-tolerant antibacterial activity against gram-positive bacteria and was found to be non-toxic to mice upon injection. Its activity was found to be highly dependent upon the four disulfide-bonds, similar to the hepcidins and defensins [11]. Thus, except for its large size, this peptide appears to have many favorable properties as a potential therapeutic candidate.

#### 4.4.3. Hemocidin

Fragments of hemoglobin have been found to have some antimicrobial activity, and have been referred to as hemocidins [129,130,131]. In the study of the Siamese crocodile, 13 fragments of crocodile hemoglobin were found to have antimicrobial activity, including peptides similar to hemoglobin β subunit [59,60]. Recently, the hemoglobin α- and β-chains of crocodilian species (*Crocodylus siamensis*, *Alligator mississippiensis*, *Crocodylus niloticus* and *Caiman crocodilus*) have been reported [132]. The *in vivo* relevance of these peptides in the crocodile remains to be determined, but it represents a potential other class of antimicrobial peptides from reptiles that should be investigated.

#### 4.4.4. Other Peptides

Other peptides have been identified in reptiles that do not fall into the various classes of antimicrobial peptides described above. These include a short, synthetic tryptophan-rich cationic antimicrobial peptide, pEM-2 (KKWRWWLKALAKK) that was shown to have broad-spectrum bactericidal activities, and was identified as a fragment of myotoxin II, a snake venom Lys49 phospholipase A2 [133,134]. Synthetic variants of this peptide were shown to have improved antimicrobial activity, salt resistance and reduced hemolytic activity [135,136]. These peptides have led to other advances in rational design of synthetic peptides, including peptides with exclusively arginine and tryptophan residues [137].

Gomes et al isolated, but did not sequence, a small 1370 Da peptide from the venom of a pit viper (*Borthrops jaracaca*), which showed very good activity against different fungi and yeast [138].

Another group identified an antimicrobial peptide derived from *N. atra* venom, the vgf-1 peptide, and found that this peptide had significant antimicrobial activity against drug-resistant clinical strains of *Mycobacterium tuberculosis* [139].

## 5. Conclusions

Reptiles are evolutionarily ancient, found in diverse and microbially challenging environments and appear to have robust immune systems. Some reptiles, especially lizards, have unique properties such as tail regeneration. All of these features suggest that reptiles may express many interesting antimicrobial peptides. A few reptilian antimicrobial peptides have been isolated and studied which demonstrate broad-spectrum antimicrobial and antifungal activity. These include members of the cathelicidin and defensin and lysozyme class.

Antimicrobial peptides are known in three of the four orders of reptiles: the testudines, crocodilians, and the squamata. No peptides are known from the sphenodontia (tuataras), as this organism has just been sequenced [41]. Reptile neutrophils appear to have granules that contain both cathelicidin-like peptides as well as β-defensin peptides, although unlike mammals, there are no genes encoding α-defensins. β-defensin-like peptides and lysozyme are also found in reptile eggs. These peptides are expressed in wounds, such as when lizards lose their tails. Detailed study of the Chinese cobra Naja atra cathelicidin peptides has revealed smaller peptides that could be useful for therapeutic applications.

Overall, reptiles reflect the diversity of antimicrobial peptides within higher organisms. They appear to express cathelicidins and β-defensins, and express hepcidins unlike avians. Additional classes of antimicrobial peptides such as lysozyme, LEAP-2 and crotamine also appear to be highly expressed in reptiles. With the advent of high-throughput genomics, new reptilian genomes have been sequenced and their transcriptomes determined. Analysis of these sequences has revealed that there are additional genes that may encode AMPs in reptiles. Future studies may reveal the in vivo role of antimicrobial peptides in reptiles. In the face of the emerging challenges due to antibiotic resistant bacteria, new and useful molecules that could be developed into future antibiotics may potentially be found in the reptilian AMPs.
